# Effect of Intraoperative Dexmedetomidine Infusion on Postoperative Bowel Movements in Patients Undergoing Laparoscopic Gastrectomy: A Prospective, Randomized, Placebo-Controlled Study

**DOI:** 10.1097/MD.0000000000000959

**Published:** 2015-06-19

**Authors:** Jin Sun Cho, Hyoung-Il Kim, Ki-Young Lee, Ji Yeong An, Sun Joon Bai, Ju Yeon Cho, Young Chul Yoo

**Affiliations:** From the Department of Anesthesiology and Pain Medicine (JSC, K-YL, SJB, JYC, YCY); Department of Surgery (H-IK, JYA); and Anesthesia and Pain Research Institute (K-YL, SJB, YCY), Yonsei University College of Medicine, Seoul, Republic of Korea.

## Abstract

Sympathetic hyperactivation is one of the causes of postoperative ileus, which occurs frequently after abdominal surgery and adversely influences the patient's prognosis. We aimed to investigate whether dexmedetomidine (DEX) could attenuate postoperative ileus in patients undergoing laparoscopic gastrectomy.

Ninety-two patients were randomized to the control (n = 46) or DEX group (n = 46). DEX was administered at a loading dose of 0.5 μg/kg for 10 minutes, followed by an infusion rate of 0.4 μg/kg/h from insufflation of the pneumoperitoneum to the end of surgery. The primary goal was to compare postoperative bowel movements by evaluating the time to first flatus. The balance of the autonomic nervous system, duration of postoperative hospital stay, and pain scores were assessed.

The time to first flatus was shorter in the DEX group compared with the control group (67.2 ± 16.8 hours vs 79.9 ± 15.9 hours, *P* < 0.001). The low-frequency/high-frequency power ratio during pneumoperitoneum increased in the control group, compared with baseline values and the DEX group. The length of postoperative hospital stay was shorter in the DEX group compared with the control group (5.4 ± 0.7 days vs 5.8 ± 1.1 days, *P* = 0.04). Patients in the DEX group had lower pain scores and required fewer analgesics at 1 hour postoperatively.

DEX facilitated bowel movements and reduced the length of hospital stay in patients undergoing laparoscopic gastrectomy. This may be attributed to the sympatholytic and opioid-sparing effects of DEX.

## INTRODUCTION

Postoperative ileus is a frequently occurring complication after abdominal surgery. It presents as the lack of flatus and defecation, along with the inability to tolerate enteral nutrition. Although the cessation of bowel movement and delayed gastric emptying can last for a maximum of 5 days after abdominal surgery, prolonged ileus may result in increased morbidity and longer hospital stays, thus increasing hospital costs.^1,2^

Surgical trauma and the direct manipulation of intestines are the most important factors in the occurrence of postoperative ileus.^3^ In comparison with open surgeries, minimally invasive techniques, such as laparoscopy, can significantly minimize trauma and inflammation in the gastrointestinal tract, thus resulting in less pain and shorter durations of postoperative ileus and hospital stay.^4^ Despite these advantages of laparoscopic surgery, pneumoperitoneum, which is required for adequate visualization during laparoscopic surgery, has been reported to induce sympathetic activation and catecholamine release.^5^ Patients undergoing laparoscopic surgery with pneumoperitoneum showed larger hemodynamic changes and sympathetic hyperactivation compared with those undergoing laparoscopic surgery with the abdominal wall-lifting method.^6^

Sympathetic hyperactivity is an important factor in the development of postoperative bowel atonia. The administration of a sympatholytic agent or epidural blockade has been reported to stimulate depressed gut motility and shorten the time to first flatus after abdominal surgery.^7,8^ Dexmedetomidine (DEX) is a highly selective α2 adrenoceptor agonist that possesses hypnotic, sedative, and sympatholytic properties without respiratory depression.^9^ Its sympatholytic effect maintains hemodynamic stability by reducing norepinephrine release.^10^

We hypothesized that DEX could attenuate sympathetic hyperactivation during laparoscopic surgery and decrease postoperative ileus. The aim of this study was to investigate the effect of intraoperative DEX administration on postoperative bowel movements in patients undergoing laparoscopic gastrectomy.

## METHODS

This randomized, double blind, placebo-controlled study was approved by the institutional review board and hospital research ethics committee of Severance Hospital at the Yonsei University Health System in Seoul, South Korea, on May 26, 2014 (No. 4-2014-0249). This study was registered at www.clinicaltrials.gov on June 9, 2014 (NCT02164448). Written consent was obtained from all of the patients. Patients (20–70 years old) who were scheduled for laparoscopic gastrectomy and had the physical status classification of I to III of the American Society of Anesthesiologists were included. Patients were excluded if they met at least one of the following criteria: heart block greater than the first degree, bradycardia (heart rate [HR] < 60 bpm), clinically significant cardiovascular, renal, or hepatic diseases, and

an allergy to α2 adrenergic agonists.

Enrolled patients were randomly assigned to either the control or the DEX group in a 1:1 ratio by using a computer-generated random number table. Assignments were concealed in sealed envelopes. The randomization was not stratified or blocked. The study drugs were prepared in identical 50-mL syringes by an anesthesia nurse who was blinded to the group assignment. DEX 200 μg was added to saline to achieve a total volume of 50 mL, resulting in a concentration of 4 μg/mL. The DEX group received the DEX infusion at a rate of 0.4 μg/kg/h after a loading dose of 0.5 μg/kg for 10 minutes. The control group received saline instead of DEX. The infusion was started after insufflation of the pneumoperitoneum and continued until the end of surgery. The surgeon, patients, attending anesthesiologists, operating room (OR) nurses, recovery nurses, and ward nurses were blinded to the group assignment.

In the OR, patients were premedicated with glycopyrrolate 0.2 mg to reduce oral secretion. Each patient was monitored using electrocardiography (EKG), pulse oximetry, noninvasive blood pressure measurements, and capnography. Oxygen saturation, HR, and mean blood pressure (MBP) were recorded every 5 minutes. After obtaining a baseline measurement of HR and MBP, anesthesia was induced with propofol 1.5 to 2.5 mg/kg and remifentanil 1 μg/kg. Following the loss of consciousness, rocuronium 0.6 mg/kg was administered to facilitate tracheal intubation. After induction, end-tidal desflurane concentrations were maintained at 4 to 6 vol%, and remifentanil was infused at a rate of 0.05 to 0.1 μg/kg/min. The anesthetic agents were titrated to maintain MBP and HR within 25% of baseline values and to provide adequate depth of anesthesia. Anesthetic depth was monitored using a bispectral index (BIS) monitor (Aspect A-2000; Aspect Medical System Inc, Newton, MA), and BIS scores were maintained in the range of 40 to 60. Controlled ventilation was performed with an oxygen–air mixture (FiO_2_ 0.4) to maintain end-tidal CO_2_ (ETCO_2_) at 35 to 40 mm Hg, and a positive end-expiratory pressure of 5 cm H_2_O was applied to all patients. Body temperature was maintained at 36 to 37 °C using a forced air warming system. Pneumoperitoneum was induced by insufflation of CO_2_, and the intra-abdominal pressure was maintained at 12 to 15 mm Hg. When the laparoscope was withdrawn, ramosetron 0.3 mg was intravenously given to prevent postoperative nausea and vomiting. The residual neuromuscular block was reversed with neostigmine 40 μg/kg and glycopyrrolate 5 μg/kg. For postoperative pain control, a patient-controlled analgesia (PCA) delivery system was started from 30 minutes before the end of surgery. The PCA system was programmed to deliver fentanyl with a basal infusion rate of 0.3 μg/kg/h and 0.15-μg/kg boluses on demand with a lockout interval of 15 minutes. After emergence from anesthesia, patients were intravenously given fentanyl 50 μg/kg to control acute pain.

The primary goal of this study was to evaluate the effect of intraoperative DEX infusion on postoperative bowel movements. The recovery of bowel function was evaluated by time to first flatus and time to first diet intake. The secondary outcomes were hemodynamic variables, heart rate variability (HRV; measure of the balance of the autonomic nervous system [ANS]), postoperative pain scores, duration of postoperative hospital stay, and complications.

Hemodynamic values were collected at baseline (T0), 10 minutes after induction and intubation (T1), 10 minutes after CO_2_ insufflation (T2), 1 hour after CO_2_ insufflation (T3), 2 hours after CO_2_ insufflation (T4), and 10 minutes after CO_2_ desufflation (T5). To evaluate ANS balance, a standard, real-time, automated, 3-lead EKG was continuously recorded with a data acquisition system to analyze HRV. The spectral analysis of HRV is widely used as a noninvasive method to assess cardiac sympathetic and parasympathetic nervous system functions, which are measured as fluctuations in R–R intervals on EKGs.^11^ HRV was analyzed using LabChart Pro version 7 with HRV modules (ADInstruments, Co., Sydney, Australia). Five-minute segments of data without ectopic beats or artifacts were analyzed from T1 to T5. Frequency-domain HRV indices were obtained with power spectral density analyses by the fast Fourier transformation. Two major power spectrum components were obtained: high-frequency (HF 0.15–0.4 Hz) power and low-frequency (LF 0.04–0.15 Hz) power. HF power represented parasympathetic nervous system activity, whereas LF power represented both sympathetic and parasympathetic nervous system activities. The ratio of LF/HF power was calculated to evaluate ANS balance. Pain scores were assessed using a numerical rating scale (NRS, 0 = no pain to 10 = worst pain) at postoperative 30 minutes, 6 hours, 24 hours, and 48 hours. Patients reporting an NRS pain score >4 at 2 consecutive evaluations were administered fentanyl 50 μg or pethidine 25 mg. Pethidine was given if shivering was also present in the postanesthesia care unit (PACU). Additional pethidine was available to both groups for breakthrough pain in the ward.

### Statistical Analysis

The sample size was calculated based on results that were previously published in patients undergoing laparoscopic gastrectomy. The average time to the return of bowel movement was 3.3 ± 0.7 days.^12^ We estimated that 42 patients in each group would be required to detect a reduction of 0.5 days with 90% power at a significance of *P* < 0.05. We factored in a 10% dropout rate and enrolled 46 patients in each group.

Statistical analyses were performed with IBM SPSS 20.0 (IBM Corp, Armonk, NY) and SAS 9.2 (SAS Institute Inc, Cary, NC). Data are shown as the number of subjects, mean ± standard deviation, or mean ± standard deviation of the mean. Comparisons between groups were performed using the χ^2^ test for categorical variables. Variables with repeated measures, such as MBP and HR, were analyzed using a linear mixed model with patient indicator as a random effect and group, time, and group-by-time as fixed effects. The group-by-time interaction assesses whether the change over time differs between groups. Between-group comparisons of continuous variables other than those previously mentioned were performed by the Student 2-sample *t* test. Post hoc analyses with the Bonferroni correction were performed for multiple comparisons when variables with repeated measures showed significant differences between groups. HRV data were tested for the normality of distribution with the Kolmogorov–Smirnov test, and data with nonnormal distributions were analyzed by the Mann–Whitney *U* test with *P* values that were adjusted by the Bonferroni correction (*P* < 0.05) for multiple comparisons among groups. A *P* value <0.05 was considered statistically significant.

## RESULTS

Ninety-two patients were initially enrolled in this study from June 14, 2014, to November 15, 2014. Two patients in the DEX group were eliminated due to conversions to open gastrectomy or concurrent cholecystectomy. The remaining 90 patients successfully completed the study without any complications. Patient characteristics were comparable between the control and the DEX groups. The durations of anesthesia and CO_2_ pneumoperitoneum were not significantly different between groups (Table 1).

**TABLE 1:**
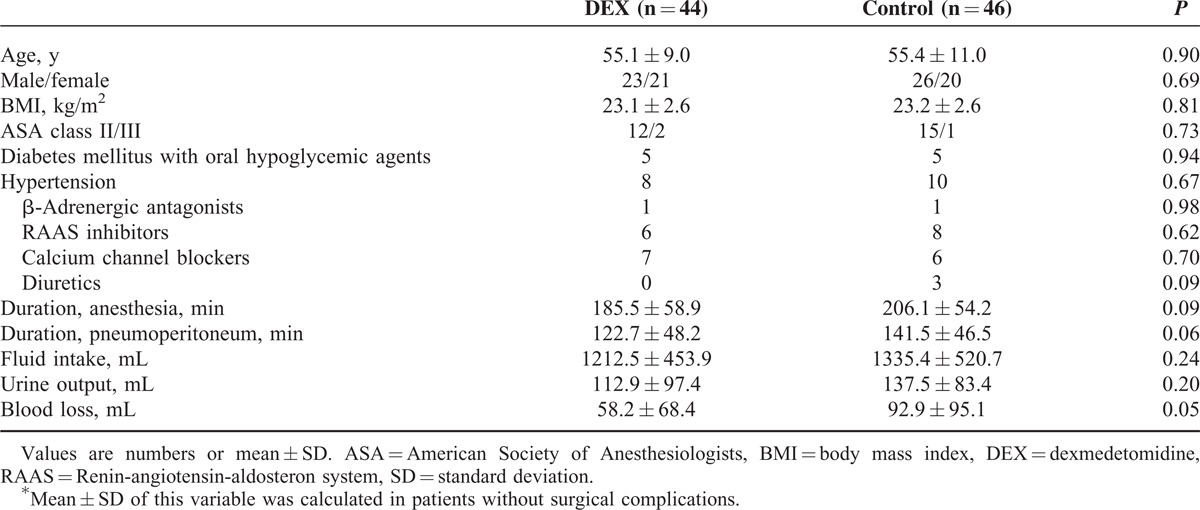
Patient Characteristics and Intraoperative Variables

### Postoperative Outcomes

The time to first flatus was earlier in the DEX group compared with the control group (67.2 ± 16.8 hours vs 79.9 ± 15.9 hours, *P* < 0.001). There were no significant differences in the time to first diet intake (3.5 ± 0.5 days vs 3.6 ± 0.6 days, *P* = 0.28) and the length of postoperative hospital stay (5.8 ± 2.0 days vs 6.8 ± 3.3 days, *P* = 0.10) between groups. However, the length of postoperative hospital stay among patients without surgical complications was significantly shorter in the DEX group compared with the control group (5.4 ± 0.7 days vs 5.8 ± 1.1 days, *P* = 0.04) (Table 2).

**TABLE 2:**
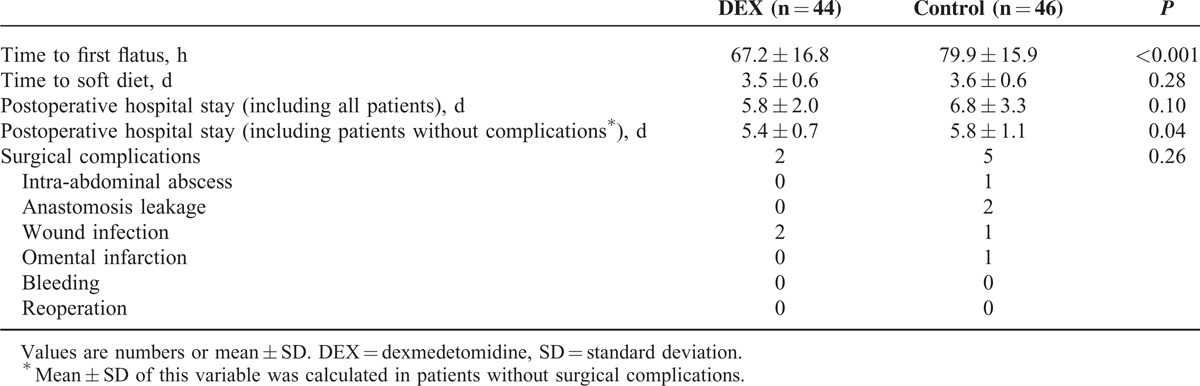
Postoperative Outcomes

### Vital Signs, ETCO_2_, BIS Scores, and Anesthetic Concentrations

Significant differences in MBP and HR were found between groups using the linear mixed model analysis (*P* = 0.002 and *P* < 0.001, respectively). Post hoc analyses with the Bonferroni correction revealed that MBP was significantly lower in the DEX group than in the control group at T3, T4, and T5. HR was significantly lower in the DEX group at T2, T3, T4, and T5. ETCO_2_, BIS, and end-tidal desflurane concentrations were similar between groups throughout all time points (Table 3). However, the concentration of remifentanil during surgery was significantly lower in the DEX group compared with the control group (0.03 ± 0.02 μg/kg/min vs 0.07 ± 0.02 μg/kg/min, *P* < 0.001).

**TABLE 3:**
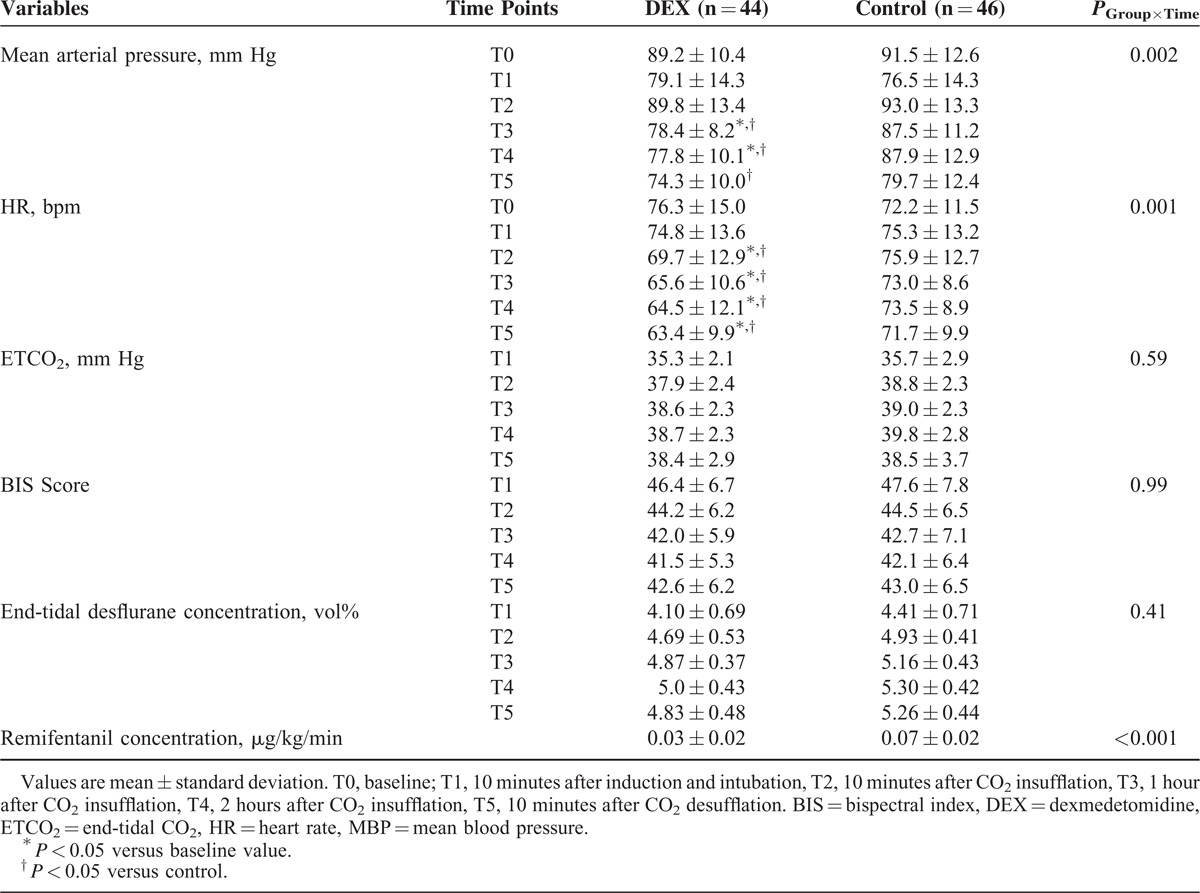
Vital Signs, ETCO_2_, BIS Scores, and Anesthetic Concentrations

### HR Variability

**FIGURE 1:**
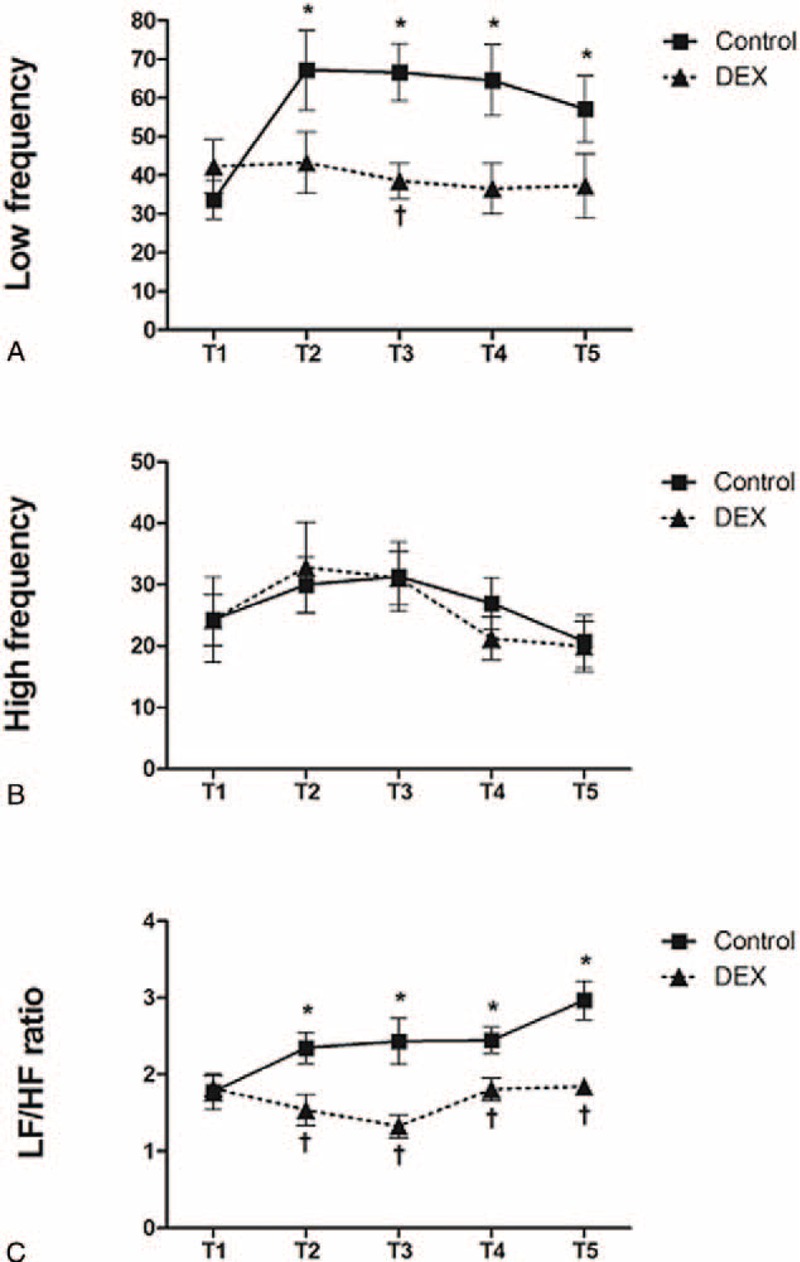
Changes in LF, HF, and the LF/HF ratio. Data are expressed as mean ± standard error of the mean. T1, 10 minutes after induction and intubation; T2, 10 minutes after CO_2_ insufflation; T3, 1 hour after CO_2_ insufflation; T4, 2 hours after CO_2_ insufflation; T5, 10 minutes after CO_2_ desufflation. *P* < 0.05 versus baseline; ^†^*P* < 0.05 versus control group. DEX = dexmedetomidine, HF = high frequency, LF = low frequency.

The LF power, HF power, and LF/HF ratio were comparable between groups at T1. In the control group, LF powers were significantly increased at T2, T3, T4, and T5, as compared with that at T1 (*P* < 0.05 for all comparisons). However, no increases in LF power were observed in the DEX group. HF powers were comparable at all time points in both groups. The LF/HF ratios in the control group were significantly increased at T2, T3, T4, and T5, as compared with that at T1 (*P* < 0.05 for all comparisons). In contrast, no increases in LF/HF ratios were observed in the DEX group. In addition, the LF/HF ratios in the DEX group were significantly lower than those in the control group at T2, T3, T4, and T5 (*P* < 0.05 for all comparisons) (Figure 1).

### Pain

NRS pain scores (3.8 ± 1.3 vs 4.7 ± 1.1, *P* = 0.001) and the number of patients requiring additional fentanyl (23/44 vs 36/46, *P* = 0.009) in the PACU was significantly lower in the DEX group vs control group. However, pain scores and the number of patients requiring “rescue” analgesics during the later postoperative time points (1–6, 6–24, and 24–48 hours) were not different between groups (Table 4).

**TABLE 4:**
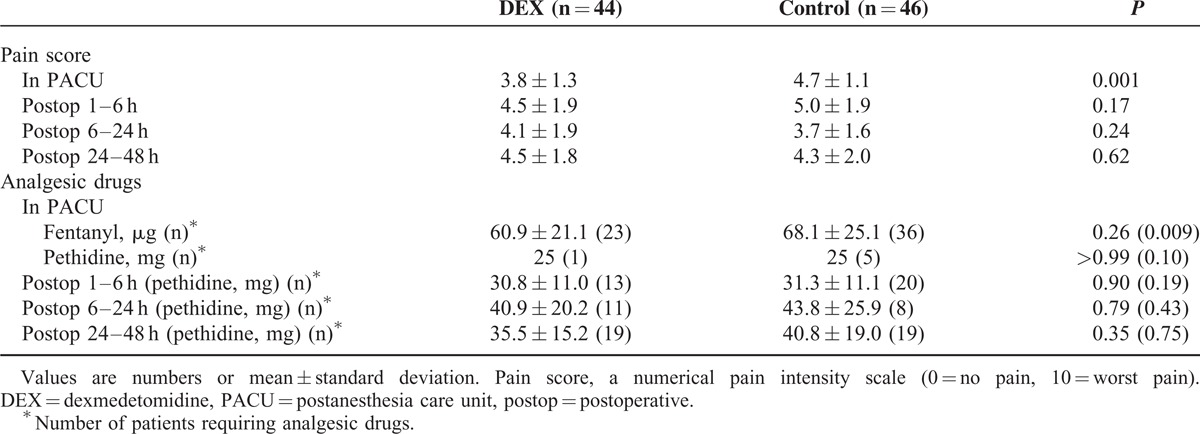
Pain Scores and Additional Analgesic Requirements

## DISCUSSION

The intraoperative administration of DEX during laparoscopic gastrectomy facilitated the early recovery of bowel function. It significantly shortened the time to first flatus and reduced the length of postoperative hospital stay. In addition, DEX reduced pain and the requirement for additional opioids during the early postoperative periods. These results might be attributed to the ability of DEX to attenuate sympathetic hyperactivation and provide analgesia.

### Pathogenesis of Postoperative Ileus: Sympathetic Activation

Postoperative ileus is a major contributor to postoperative morbidity and prolonged convalescence or hospital stay after major abdominal surgery. The pathophysiology of postoperative ileus is complex and involves many factors, including surgical trauma, activation of inhibitory sympathetic reflexes, and induction of local and systemic inflammatory mediators.^1^ In comparison with open surgery, laparoscopic gastrectomy has been reported to reduce surgical trauma and attenuate the immune response, thus reducing the time to flatus and the time to the resumption of a soft diet.^4,13^ However, the induction of pneumoperitoneum and surgical stimuli for laparoscopic visualization can cause sympathetic activation. Parasympathetic stimulation increases gastrointestinal motility, whereas sympathetic stimulation serves as the predominant inhibitory impetus to the bowel. Pneumoperitoneum reduces venous return and decreases cardiac output, which might provoke the baroreceptor reflex and sympathetic activation when combined with the reversed Trendelenburg position.^5,14^ High-pressure pneumoperitoneum shows greater increases in sympathetic activity than low-pressure pneumoperitoneum.^15^ In addition, CO_2_ pneumoperitoneum can induce hypercarbia, which directly and indirectly stimulates the sympathetic nervous system by increasing catecholamine levels.^16^ These sympathetic activation and inhibitory inputs have been demonstrated to be factors in the development of postoperative ileus.^17,18^ Furthermore, the mechanism of sympathetic inhibition involves preventing the release of acetylcholine, which is essential for intestinal peristalsis, from excitatory fibers that are located in the myenteric plexus.^19^

### Previous Efforts to Attenuate Sympathetic Activation

In an effort to reduce ileus, epidural anesthesia or the administration of adrenoblocking agents has been used for sympathetic blockade. Dihydroergotamine, which acts as a sympatholytic agent in the gastrointestinal tract, reduces the time to first bowel movement and increases bowel movements, as determined via electromyography.^7^ Epidural blockade has been proven to improve postoperative ileus, and its effect may be associated with the blockade of afferent and efferent inhibitory reflexes, as well as efferent sympathetic blockade.^8^ However, conflicting results regarding the duration of blockade, drug administration, and level of blockade have been reported. To this date, the administration of adrenoblocking agents or parasympathetic agonists has not effectively reduced postoperative ileus.

### DEX: Sympatholytic Effects

DEX is a highly selective α2 adrenoceptor agonist that has sympatholytic and analgesic effects. It acts on central α2 adrenoceptors to reduce sympathetic tone and decrease catecholamine levels.^20^ In addition, α2 adrenoceptor agonists have been reported to induce nitric oxide-dependent vasorelaxation that is mediated by endothelial α2 adrenoceptor activation.^21^ The ability of DEX to attenuate sympathetic activation when used as a sedative or adjunct to general anesthesia has been proven by measuring HRV.^22,23^ The spectral analysis of HRV is widely used as a noninvasive method to assess cardiac sympathetic and parasympathetic nervous system functions.^24^ In this study, during operations, the LF/HF ratio was used to measure the balance between sympathetic and parasympathetic tones in the ANS and was significantly increased in the control group compared with baseline values. This represented sympathetic hyperactivation. In contrast, the LF/HF ratio in the DEX group was maintained at baseline levels during operations. This suggests that DEX attenuates pneumoperitoneum- and surgical stress-induced sympathetic activation. These findings are consistent with the results of previous studies that investigated the effects of DEX on autonomic responses during stressful events. DEX has been shown to blunt the sympathetic effect of sweating and attenuate the shivering-induced increase in blood pressure and catecholamine release.^22^ It also suppresses sympathetic hyperactivation during endotracheal intubation.^23^

However, DEX has the potential to cause hypotension due to systemic vasodilation via sympatholytic actions.^25^ It usually elicits a biphasic hemodynamic response with an initial increase in blood pressure and reflex bradycardia, followed by a subsequent return to baseline after stabilization.^26^ Patients in the DEX group had low blood pressures and HR, which may be due to the sympatholytic effect of DEX. However, the chosen dose in this study trial (0.4 μg/kg/h) appeared to be safe, as blood pressures and HR were maintained within 25% of baseline values. An increase in the relative risk of developing bradycardia and hypotension (requiring treatment) was reported only after administering a loading dose and maintenance dose of >0.7 μg/kg/h DEX in critically ill patients.^27^

### DEX: Analgesic and Opioid-Sparing Effects

Adequate pain relief can contribute to the reduction of postoperative ileus by allowing the patients to be mobilized earlier. Therefore, enteral feeding can be instituted. Although opioids are preferred for postoperative pain control, they are notorious for effects on inhibiting gastrointestinal motility and aggravating postoperative ileus.^28,29^ In this study, the intraoperative use of DEX reduced opioid consumption during surgery and the requirement of rescue fentanyl during the postoperative 1-hour period. The DEX group had lower pain scores, and less patients required rescue analgesics (fentanyl) in the PACU. Previous studies also reported a reduction in opioid requirements as well as decreased postoperative pain^30,31^ and catheter-related bladder discomfort^32^ after intraoperative DEX administration. The opioid-sparing effects of DEX may help to reduce the opioid-induced inhibition of gastrointestinal motility. The failure of DEX to produce a sustained opioid-sparing effect during the later postoperative period in our study might be related to its short elimination half-life of 2 hours. Because the time course of the stress response to surgery continues beyond the operative period, intraoperative DEX administration may only modify the stress response for a relatively short period of time. The continuous infusion of DEX during postoperative periods is likely to result in better analgesia and earlier recovery of bowel function. Therefore, further research on the administration of DEX during perioperative periods is warranted.

## LIMITATIONS

The mechanisms by which DEX improves bowel recovery may involve intraoperative sympatholysis, which reduces ileus by attenuating sympathetic inhibitory impetus to the bowel, and the reduction of systemic opioids, which induces ileus by activating μ-receptors in the gastrointestinal tract. Both mechanisms may contribute to the reduction of ileus, although the impact of each mechanism on the return of bowel movement is unknown. In addition, the durations of anesthesia and pneumoperitoneum were relatively shorter in the DEX group than in the control group, although there were no statistical differences between the groups. There is a possibility that they might affect the postoperative outcomes. Last, no statistically significant differences in the length of postoperative hospital stay were observed between groups when all patients were included in the analysis. We believe that the benefits of DEX could not have overcome any delays in recovery that were caused by surgical complications. However, when patients with major surgical complications were excluded from the analysis, the length of postoperative hospital stay was significantly reduced in the DEX group. Further studies are needed to investigate this finding.

## CONCLUSION

The administration of DEX during laparoscopic gastrectomy facilitated the early recovery of bowel function after surgery. Our findings suggest that DEX can maintain autonomic balance by attenuating the pneumoperitoneum- and surgery-induced hyperactivation of the sympathetic nervous system.
